# Effects of a single dose of *N*-Acetyl-5-methoxytryptamine (Melatonin) and resistance exercise on the growth hormone/IGF-1 axis in young males and females

**DOI:** 10.1186/1550-2783-4-14

**Published:** 2007-10-23

**Authors:** Erika Nassar, Chris Mulligan, Lem Taylor, Chad Kerksick, Melyn Galbreath, Mike Greenwood, Richard Kreider, Darryn S Willoughby

**Affiliations:** 1 Department of Health, Human Performance, and Recreation, Baylor University, Box 97313, Waco, TX 76798, USA; 2 Department of Nutrition and Food Science, Colorado State University, Fort Collins, CO 80523, USA; 3 Department of Health, Leisure, and Exercise Science, University of West Florida, Pensacola, FL 32514, USA; 4 Department of Health and Exercise Science, University of Oklahoma, Norman, OK 73019-6081, USA; 5 Institute for Biomedical Studies, Baylor University, Waco, TX 76798, USA

## Abstract

Melatonin and resistance exercise alone have been shown to increase the levels of growth hormone (GH). The purpose of this study was to determine the effects of ingestion of a single dose of melatonin and heavy resistance exercise on serum GH, somatostatin (SST), and other hormones of the GH/insulin-like growth factor 1 (IGF-1) axis. Physically active males (n = 30) and females (n = 30) were randomly assigned to ingest either a melatonin supplement at 0.5 mg or 5.0 mg, or 1.0 mg of dextrose placebo. After a baseline blood sample, participants ingested the supplement and underwent blood sampling every 15 min for 60 min, at which point they underwent a single bout of resistance exercise with the leg press for 7 sets of 7 reps at 85% 1-RM. After exercise, participants provided additional blood samples every 15 min for a total of 120 min. Serum free GH, SST, IGF-1, IGFBP-1, and IGFBP-3 were determined with ELISA. Data were evaluated as the peak pre- and post-exercise values subtracted from baseline and the delta values analyzed with separate three-way ANOVA (p < 0.05). In males, when compared to placebo, 5.0 mg melatonin caused GH to increase (p = 0.017) and SST to decrease prior to exercise (p = 0.031), whereas both 0.5 and 5.0 mg melatonin were greater than placebo after exercise (p = 0.045) and less than placebo for SST. No significant differences occurred for IGF-1; however, males were shown to have higher levels of IGFBP-1 independent of supplementation (p = 0.004). The 5.0 mg melatonin dose resulted in higher IGFBP-3 in males (p = 0.017). In conclusion, for males 5.0 mg melatonin appears to increase serum GH while concomitantly lowering SST levels; however, when combined with resistance exercise both melatonin doses positively impacts GH levels in a manner not entirely dependent on SST.

## Background

Growth hormone is secreted from the adenohypohysis of the pituitary gland and is regulated by a final common pathway, consisting of inhibitory control of somatostatin (SST) and stimulatory control of hypothalamic GH-releasing hormone (GHRH). As such, GH is known to display profound anabolic effects at the tissue level and has been implicated to play a significant role in metabolic regulation, protein synthesis, and cell multiplication and differentiation in skeletal muscle. Once secreted into the circulation, free, unbound GH has a half-life of approximately nine minutes [1]. Almost 50% of GH in circulation is bound to a high-affinity GH binding protein (GHBP), and GHBP seems to directly correlate with tissue GH receptor content [2]. However, it is the free, unbound GH that is able to bind to the extracellular domain of the GH receptor, thereby effecting GH kinetics which can invariably regulate growth factor release and GH signaling in skeletal muscle. Relative to skeletal muscle, the anabolic role of GH is based on the circulating levels of free GH and exists due to its influence on the activity of various growth factors such as insulin-like growth factor 1 (IGF-1).

The majority of GH secretion from the pituitary is controlled by hormonal signals received from the hypothalamus. In addition, GH release can also be regulated at the hypothalamic level by various metabolic stress signals such as acute resistance exercise. The release of GH has been shown to occur in response to single bouts of resistance exercise. A single bout of resistance exercise at 85% of the one-repetition maximum (1-RM) was shown to significantly elevate serum GH [3]. Also, when compared to a single set of high-intensity resistance exercise at 90% 1-RM, a single set of low- and moderate-intensity resistance exercise at either 50% or 70% 1-RM immediately following high intensity exercise resistance exercise at 90% 1-RM produced greater increases in serum GH levels [4]. These data suggest that a high intensity, low volume training protocol known to typically induce neural adaptation does not result in a serum GH response commensurate with those observed to occur with low- to moderate-intensity resistance exercise.

The indolamine melatonin (*N*-Acetyl-5-methoxytryptamine) is a lipophilic hormone synthesized by the pineal gland and has been shown to be involved in hormone release [5]. As such, melatonin has been shown to play a facilitatory role in the neuroregulation of growth hormone (GH) secretion at the hypothalamic level, evidenced by peak elevations in serum GH in males 60 min after the acute oral ingestion of melatonin at doses of 500 mg [6] and 12 mg [7]. It has been suggested that the mechanism for melatonin's neuroregulatory role is through the binding of meltatonin receptors on the hypothalamus, thereby inhibiting SST release [8]. This mechanism has been further corroborated in a study where 10 g of oral melatonin provided to males elevated basal GH release and GH responsiveness to GHRH at 60 and 120 min after ingestion, while concomitantly inhibiting endogenous SST release [9]. These data lend support to the contention that melatonin supplementation increases the activity of the SST receptor-effector system. Since exercise-induced GH secretion is thought to be mediated predominantly through a hypothalamic pathway, it seems likely that melatonin facilitates GH secretion at a hypothalamic level. Exogneous oral melatonin given at a dose of 5.0 mg prior to bicycle exercise at 70% VO_2max _was shown to cause significant increases in serum GH levels and those of IGF binding protein-1 (IGFBP-1) when compared to placebo [10]. However, 6.0 g of oral melatonin was shown to have no effect on serum GH levels for 60 min following a single bout of resistance exercise at 70%–85% 1-RM [11].

The primary purpose of this study was to determine the effects of either a placebo or a melatonin supplement administered at a dose of either 0.5 mg or 5.0 mg prior to a single bout of heavy-resistance leg press exercise on free GH, IGF-1, IGFBP-1, IGF binding protein-3 (IGFBP-3), and SST levels in young men and women. A secondary purpose was to assess the safety profiles of acute melatonin supplementation by way of evaluating the systemic hemodynamic effects and serum and whole blood clinical markers in response to the three separate supplementation protocols.

## Methods

### Participants and screening

Sixty apparently healthy, resistance-trained (regular resistance training at least twice weekly for at least one year) males [n = 30 (22.72 ± 3.28 years, 180.94 ± 6.24 cm, 82.08 ± 9.18 kg)] and eumenorrheic females [n = 30 (21.90 ± 2.85 years, 165.91 ± 5.81 cm, 62.43 ± 8.50 kg)] served as participants in the study. All participants were cleared for participation by passing a mandatory medical screening. Only participants considered as either low or moderate risk and with no contraindications to exercise as outlined by the American College of Sports Medicine (ACSM) and/or who had not consumed any nutritional supplements (excluding multi-vitamins) 2 months prior to the study were allowed to participate. Additionally, participants were not eligible if they had taken any over-the-counter or prescription anti-inflammatory medication within two weeks prior to the study. All eligible participants were asked to provide oral and informed written consent based on university-approved documents and approval was granted by the Institutional Review Board for Human Subjects of Baylor University. Additionally, all experimental procedures involved in the study conformed to the ethical consideration of the Helsinki Code.

### Entry/familiarization and baseline strength testing session

Participants believed to meet eligibility criteria were invited to attend an entry/familiarization and baseline strength testing session. Prior to this, participants were instructed to refrain from lower-body resistance exercise for 48 hours prior to baseline testing. Participants meeting entry criteria were familiarized to the study protocol by way of a verbal and written explanation outlining the study design. Participants were then subjected to an initial strength test to assess their one repetition maximum (1-RM) on the leg press exercise (Nebula, Versailles, OH) to be used in the study. Heart rate and blood pressure were monitored throughout the strength testing session. Once the 1-RM was determined, participants were asked to perform and practice the proposed resistance exercise session in its entirety (see section for "Training and Supplementation") without blood sampling to familiarize them with the protocol and to also insure that they were able to complete the protocol before being formally admitted to the study. At the conclusion of the entry/familiarization and baseline strength testing session, each participant meeting the eligibility criteria was given an appointment time one week later to begin the study.

### Resistance exercise protocol and supplementation

Male and female participants were matched by relative strength levels (strength ÷ body weight) and then randomly assigned to one of three different supplement protocols. For the supplement protocol, in a double-blind fashion, groups received either 0.5 mg or 5.0 mg of *N*-Acetyl-5-methoxytryptamine (melatonin) or 1.0 mg of a dextrose placebo. Following the study protocol established previously [10], each participant underwent a testing session with a total duration of 180 minutes that began at 2:00 pm. The participants were all provided with a light breakfast at 8:00 am consisting of a meal replacement bar (Premier Nutrition, Carlsbad, CA) containing 7 g of fat, 25 g of carbohydrate, 30 g of protein, and 280 kilocalories, and were then required to fast until 2:00 pm. Seventy-five minutes prior to each exercise session (12:30 pm), an intravenous cannula was inserted into a forearm vein. Sixty minutes prior to the exercise session (-60), a baseline blood sample was taken and then participants orally received a placebo or melatonin supplement (Iovate Health Sciences, Inc.) at a dosage or either 0.5 or 5.0 mg. During this 60-minute period of time, participants rested in a supine position and blood samples were obtained every 15 minutes up to the initiation of the resistance exercise trial (-45, -30, -15, 0). Using the resistance exercise protocol previously established [3], participants performed the leg press exercise consisting of 7 sets of 7 repetitions at 85% 1-RM; each set was performed over the course of 30 seconds and followed by 150 seconds of rest. As such, the resistance exercise session involved a total duration of 20 minutes.

**Figure 1:**
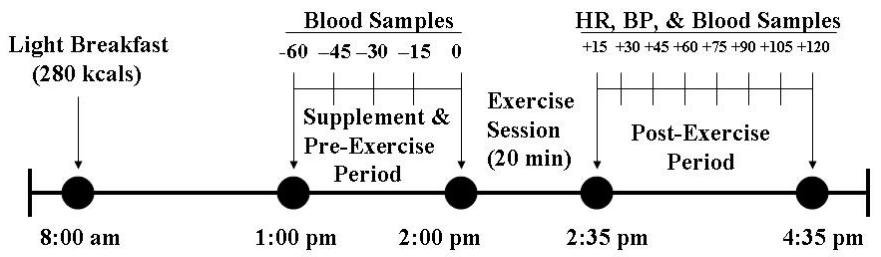
An illustration of the experimental protocol used in the study.

Heart rate and blood pressure were assessed during the rest period between each set. A blood sample was obtained 15 minutes following the completion of the exercise session and at 15-minute intervals such that the last blood sample was obtained at 120 minutes following exercise (+15, +30, +45, +60, +75, +90, +105, +120). Additionally, heart rate and blood pressure were assessed at each blood sampling point. Each participant remained recumbent and awake during the 120-minute post-exercise period (see Figure 1 for an illustration of the experiment protocol).

### Dietary records

Food intake was not standardized prior to the study; however, participants were required to do a dietary recall for the 48 hours prior to the resistance exercise session. The 48-hour dietary recalls were evaluated with the Food Processor dietary assessment software program (ESHA Research, Salem, OR) to determine the average daily macronutrient consumption of fat, carbohydrate, and protein in the diet prior to supplementation and exercise.

### Blood collection procedures

Venous blood samples were obtained into 10 ml vacutainer tubes from a 20 gauge intravenous catheter inserted into a forearm vein after the subcutaneous injection of a topical anesthetic (2% Xylocaine). Following a five hour fast, blood samples were obtained at 15-minute intervals 60 minutes prior to the resistance exercise session and then at 15-minute intervals for 120 minutes following the resistance exercise session. Blood samples were allowed to stand for 10 minutes at room temperature and then centrifuged at 2,400 rpm for 10 minutes. Serum was then separated and stored at -80°C until analysis.

### Clinical chemistry analyses

Serum samples were analyzed for clinical chemistry markers (i.e., glucose, total protein, blood urea nitrogen, creatinine, BUN/creatinine ratio, AST, ALT, CK, LDH, GGT, albumin, globulin, sodium, chloride, calcium, carbon dioxide, total bilirubin, alkaline phosphatase, triglycerides, cholesterol, HDL, LDL) using a Dade Dimension RXL clinical chemistry analyzer (Dade-Behring Inc., Newark, DE). Whole blood samples were analyzed for standard cell blood counts with percentage differentials (i.e., hemoglobin, hematocrit, erythrocyte counts, MCV, MCH, MCHC, RDW, leukocyte counts (neutrophils, lymphocytes, monocytes, eosinophils, basophils) using an Cell-Dyn 3500 hematology analyzer (Abbott Laboratories Inc., Pasadena, CA).

### Serum hormone analyses

Using enzyme-linked immunoabsorbent assays (ELISA) and enzyme-immunoabsorbent assays (EIA), serum samples were assayed for free GH, IGF-1, IGFBP-1, IGFBP-3, (Diagnostics Systems Laboratories, Webster, TX), and SST (Bachem, Torrance, CA) with a Wallac Victor-1420 microplate reader (Perkin-Elmer Life Sciences, Boston, MA), and the assays were performed in duplicate at a wavelength or either 450 or 405 nm, respectively. For GH, an ultra-sensitive assay with a sensitivity of 0.66 pg/ml was used to determine the levels of free, unbound GH without the detectable interference of circulating glycoproteins, GH-dependent peptide hormones, or GHBP. For IGF-1, an ultra-sensitive assay with a sensitivity of 0.015 ng/ml was used to detect free IGF-1, with no cross-reactivity with IGF-II or IGFBPs. The assays for IGFBP-1, IGFBP-3, and SST had sensitivities of 0.25 ng/ml, 0.04 ng/ml, and 0.06 ng/ml, respectively. The average (± SD) coefficients of variation for the duplicates of all samples performed for each hormone were 6.65 ± 1.45%, 7.34 ± 1.67%, 7.12 ± 1.75%, 8.73 ± 2.02%, and 7.23 ± 2.85%, respectively, for GH, IGF-1, IGFBP-1, IGFBP-3, and SST.

### Assessment of hemodynamic safety markers (heart rate and blood pressure)

At each blood sampling period, participants underwent the assessment of hemodynamic safety markers (heart rate and blood pressure). Heart rate was determined by use of a Polar heart rate monitor (Polar, San Ramon, CA), and blood pressure was assessed in the supine position using a mercurial sphygmomanometer using standard procedures.

### Statistical analyses

Statistical analyses were performed by utilizing separate 3 × 2 × 2 [Group (placebo, 0.5 mg, 5.0 mg] × Gender (male, female) × Test (pre-exercise, post-exercise) factorial analyses of variance (ANOVA) with repeated measures for each criterion variable. Pair-wise comparisons were used to differentiate significant differences among the three factors. All statistical procedures were performed using SPSS 11.0 software and a probability level of < 0.05 was adopted throughout.

## Results

### Resistance exercise session

The average intensity with which all the participants completed the exercise bout at the required 7 sets of 7 repetitions was 84.7% ± 0.004 of the 1-RM, and it has been shown that exercise at this intensity produces a GH response [3,4].

### Dietary analysis

Results showed that there were no significant differences in total calories or the macronutritent intakes of carbohydrates, fats, and protein between the three groups in the 48 hours prior to the testing session (p > 0.05) (data not shown).

### Serum hormones

For very short half-life hormones that are highly sensitive to diurnal variations, such as GH, we observed much variability in the baseline levels even though the testing time and food intake on each day was standardized. In addition, since we employed such a broad sampling window, we also observed much variability throughout the pre- and post-exercise periods. Therefore, peak serum hormone responses before and after resistance exercise were subtracted from the baseline values to determine delta values. For standardization purposes, all serum hormones were expressed in the same manner.

**Figure 2:**
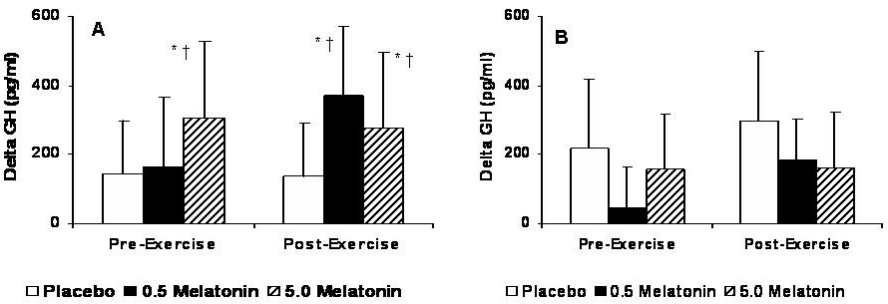
Free GH for males (A) and females (B) expressed as the delta values for the peak changes pre- and post-exercise relative to baseline values. * Significantly different from placebo (p < 0.05). † Significantly different from the corresponding values for females (p < 0.05).

For GH, a significant Group × Gender × Test interaction was observed (p = 0.012) indicating that both melatonin supplements and resistance exercise was effective in elevating serum GH levels, and that this response was more pronounced in males. In males, pair-wise comparisons revealed 5.0 mg melatonin to result in higher GH levels than placebo (p = 0.017) during the pre-exercise period while a trend was evident suggesting 0.5 mg melatonin to be greater than placebo (p = 0.077). During the post-exercise period, both melatonin doses were greater than placebo (p = 0.042). In females, a trend existed in the post-exercise period for the 0.5 mg supplement (p = 0.064) (Figure 2).

**Figure 3:**
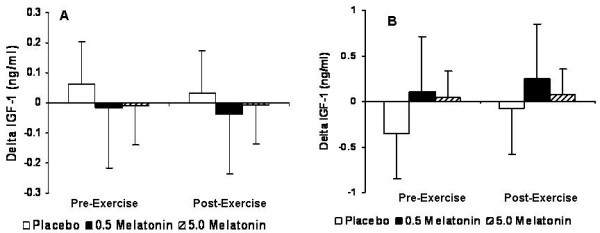
IGF-1 for males (A) and females (B) expressed as the delta values for the peak changes pre- and post-exercise relative to baseline values. There were no significant differences located for IGF-1 (p > 0.05).

**Figure 4:**
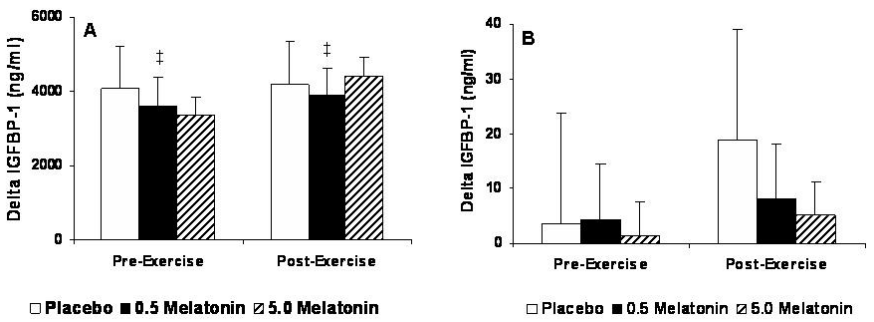
IGFBP-1 for males (A) and females (B) expressed as the delta values for the peak changes pre- and post-exercise relative to baseline values. ‡ Significantly different from each corresponding value in females (p < 0.05).

For IGF-1, no significant Group × Gender × Test interaction (p = 0.345) or main effects were noted for either gender (p > 0.05) (Figure 3). For IGFBP-1, a significant Group × Gender interaction was observed (p = 0.004) indicating that the effects of supplementation on IGFBP-1 was different between genders, but was not affected by resistance exercise. Pair-wise comparisons showed that males exhibited a greater response in IGFBP-1 than females (p = 0.022), and that the placebo (p = 0.034) and 0.5 mg melatonin (p = 0.043) were greater than 5.0 mg melatonin (Figure 4).

**Figure 5:**
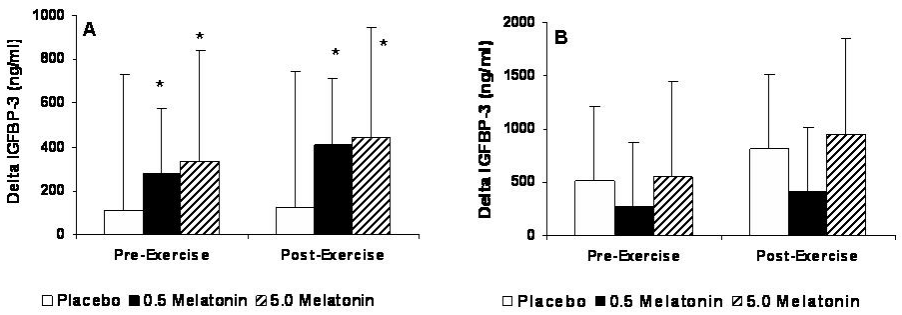
IGFBP-3 for males and females expressed as the delta values for the peak changes pre- and post-exercise relative to baseline values. * Significantly different from placebo (p < 0.05).

For IGFBP-3, a significant Group × Gender × Test interaction was not observed (p = 0.412); however, for males a main effect for Gender (p = 0.017) and Test (p = 0.028) was observed and indicated that males demonstrated a greater IGFBP-3 response than females. For males, pair-wise comparisons showed that both 0.5 mg and 5.0 mg melatonin was significantly greater than placebo in the post-exercise period (see Figure 5).

**Figure 6:**
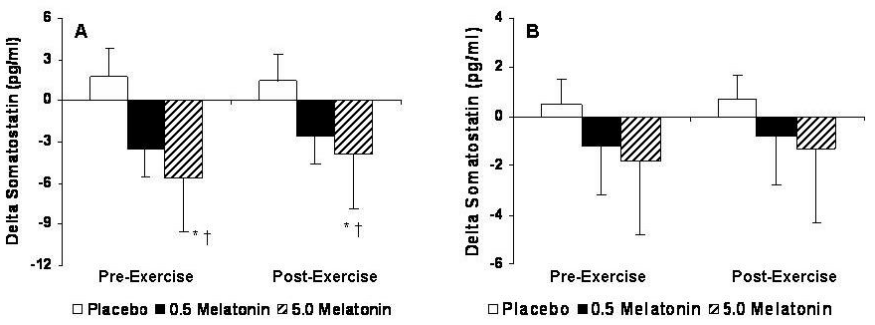
SST for males (A) and females (B) expressed as the delta values for the peak changes pre- and post-exercise relative to baseline values. * Significantly different from placebo (p < 0.05). † Significantly different from the corresponding values for females (p < 0.05).

For SST, a significant Group × Gender × Test interaction was observed (p = 0.036) indicating that both melatonin supplements and resistance exercise was effective in elevating serum GH levels, and that this response was more pronounced in males. In males, pair-wise comparisons revealed 5.0 mg melatonin to result in lower SST levels than placebo (p = 0.031) during the pre-exercise period while a trend was evident suggesting 0.5 mg melatonin to be greater than placebo (p = 0.082, effect size = 0.029). During the post-exercise period, both melatonin doses were less than placebo (p = 0.046), but not different from one another. In females, a trend existed in the pre-exercise period for the 5.0 mg supplement (p = 0.078) (Figure 6).

### Hemodynamic and clinical chemistry analyses

Results showed no significant abnormal effects on either hemodynamic or clinical chemistry variables between groups as a result of either the supplementation or the resistance exercise (p > 0.05) (Tables 1, 2, 3).

**Table 1:** Hemodynamic Clinical Safety Markers

Time	0.5 mg				Placebo				5.0 mg			
	
	-60	0	15	120	-60	0	15	120	-60	0	15	120
Heart Rate (bpm)	67 ±11.9	67 ±10.7	84 ±9.9	63 ±8.0	59 ±10.6	60 ±8.4	77 ±9.2	61 ±10.5	68 ±10.1	64 ±8.4	78 ±9.4	60 ±7.2
SBP (mmHg)	114 ±7.8	111 ±5.8	118 ±10.6	110 ±10.4	114 ±6.4	114 ±7.9	115 ±7.6	111 ±8.1	110 ±7.4	113 ±8.3	117 ±6.9	112 ±8.4
DBP (mmHg)	77 ±4.8	70 ±6.7	73 ±7.0	69 ±11.9	70 ±5.6	72 ±8.2	73 ±6.4	73 ±9.5	76 ±4.7	76 ±5.8	78 ±7.3	73 ±6.3

Data are presented as means ± SD. Results showed no significant differences between groups as a result of either the supplementation or the resistance exercise (p > 0.05).

**Table 2:** Serum Clinical Chemistry Markers

Time	0.5 mg				Placebo				5.0 mg			
	
	-60	0	15	120	-60	0	15	120	-60	0	15	120
Triglyceride (mg/dl)	75±22	67±16	63±12	63±13	76±28	76±25	76±30	83±37	83±32	80±42	74±38	70±34
Cholesterol (mg/dl)	160±28	164±30	164±35	168±36	167±24	167±38	160±34	165±37	156±20	152±19	160±22	167±28
HDL (mg/dl)	49±10	49±11	51±10	51±11	54±13	53±16	53±14	53±15	56±18	52±13	57±19	60±17
LDL (mg/dl)	95±27	95±27	96±29	98±31	97±16	98±23	94±20	96±22	82±15	84±15	84±16	88±21
GGT (U/L)	23±5.1	22±5.3	23±4.1	22±4.9	25±6.9	26±7.7	25±7.5	24±7.5	25±5.3	25±5.9	25±4.5	25±5.8
LDH (U/L)	231±134	150±51	200±188	121±16	168±51	181±81	266±283	210±83	191±108	218±263	139±22	131±15
Uric Acid (g/dl)	4.7±.71	4.8±.74	5.2±.73	5.6±.80	5.5±.88	5.3±.83	5.1±2.0	5.8±1.2	5.3±.88	5.2±.96	5.5±.87	5.7±.93
Glucose (mg/dl)	99±51	99±48	98±38	92±27	98±22	92±13	89±5.3	89±6.9	87±9.4	85±6.6	88±9.4	87±9.5
BUN (mg/dl)	15±4.8	15±4.9	15±4.1	14±4.2	15±3.9	15±4.0	14±3.5	13±2.9	16±4.8	16±4.3	15±4.6	15±3.3
Creatinine (mg/dl)	1.1±.20	1.2±.23	1.3±.29	1.2±.20	1.0±.13	1.1±.23	1.2±.21	1.1±.22	1.1±.21	1.1±.13	1.3±.17	1.1±.17
Calcium (mg/dl)	9.2±.29	9.4±.37	9.5±.41	9.6±.30	9.4±.39	9.4±.37	9.6±.45	9.6±.60	9.2±.70	9.4±.54	9.6±.52	9.6±.64
Total Protein (g/dl)	7.3±.34	7.3±.36	7.5±.46	7.4±.51	7.6±.42	7.6±.37	7.6±.61	7.5±.61	7.2±.45	7.3±.61	7.3±.48	7.4±.51
Albumin (g/dl)	4.6±.27	4.7±.30	4.7±.29	4.8±.27	4.8±.34	4.8±.31	4.7±.36	4.8±.38	4.6±.33	4.6±.27	4.6±.43	4.7±.39
Total Bilirubin (mg/dl)	.77±.25	.75±.25	.79±.28	.84±.32	.68±.24	.67±.21	.74±.25	.82±.27	.78±.42	.74±.47	.74±.46	.83±.47
ALP (U/L)	76±18	76±16	76±16	77±13	79±16	74±16	74±21	75±19	69±14	74±18	71±12	71±14
AST (U/L)	32±16	23±9	27±20	20±7	31±16	34±17	38±22	37±16	28±12	21±6.7	22±5.1	21±5.7
ALT (U/L)	26±4.7	25±4.1	26±4.7	25±5.1	28±12	30±16	30±16	28±15	27±9.8	27±11	27±9.4	28±9.3
CK (U/L)	253±273	231±257	259±298	250±253	273±363	286±370	281±351	262±326	173±105	177±109	182±102	179±101

Data are presented as means ± SD. Results showed no significant differences between groups as a result of either the supplementation or the resistance exercise (p > 0.05).

**Table 3:** Whole Blood Clinical Chemistry Markers

Time	0.5 mg				Placebo				5.0 mg			
	
	-60	0	15	120	-60	0	15	120	-60	0	15	120
WBC (cells/μl)	5.4±.84	5.4±1.1	6.3±2.1	7.2±1.8	4.5±.98	4.5±.84	5.7±2.0	6.0±1.1	6.1±2.3	6.1±2.4	6.4±2.4	7.0±2.8
RBC (cells/μl)	5.0±.26	5.0±.31	5.1±.17	5.0±.25	4.7±.43	4.7±.31	4.8±.27	4.7±.31	4.9±.28	4.8±.41	4.9±.33	4.8±.37
Hemoglobin (g/dl)	15±.60	15±.69	15±.35	15±.53	14±1.4	14±.80	14±.74	14±.96	15±.74	14±1.0	15±.78	14±.89
Hematocrit (%)	45±1.7	44±2.0	45±.97	44±1.7	42±4.2	42±2.4	43±2.0	42±2.2	43±1.9	42±3.0	43±2.4	42±2.7
MCV (fL)	89±2.1	89±2.0	89±2.1	89±2.1	89±3.0	89±3.1	89±3.4	89±3.2	88±1.7	89±1.7	88±1.5	88±1.7
MCH (pg/cell)	30±.78	30±.89	30±.72	30±.72	30±1.2	30±1.5	30±1.2	30±1.4	30±.60	30±.51	30±.70	30±.70
MCHC (g/dl)	34±.35	34±.33	34±.33	34±.50	34±.44	34±1.1	34±.55	34±.97	34±.70	34±.41	34±.56	34±.69
Neutrophils (cells/μl)	3.1±.59	3.3±.78	3.9±1.1	5.1±1.7	2.3±.79	2.3±.73	3.9±2.0	4.1±1.3	3.7±2.0	3.9±2.0	4.2±1.9	4.8±2.4
Lymphocyte (cells/μl)	1.6±.38	1.8±.83	2.1±1.3	2.1±1.8	1.8±.66	1.4±.19	1.2±.18	1.4±.33	1.6±.48	1.5±.41	1.6±.51	1.6±.47
Monocytes (cells/μl)	.43±.10	.41±.09	.45±.18	.47±.17	.32±.11	.33±.10	.35±.09	.39±.13	.50±.19	.48±.20	.49±.21	.51±.20
Eosinophils (cells/μl)	.21±.12	.18±.11	.17±.11	.14±.12	.14±.07	.12±.06	.10±.05	.09±.06	.19±.16	.19±.16	.17±.14	.13±.12
Basophils (cells/μl)	.06±.01	.07±.03	.07±.03	.06±.02	.06±.02	.06±.02	.06±.02	.06±.02	.08±.02	.06±.01	.06±.02	.07±.02

Data are presented as means ± SD. Results showed no significant differences between groups as a result of either the supplementation or the resistance exercise (p > 0.05).

## Discussion

For some of the hormones, GH in particular, we observed much variability in the baseline values, even though the participants were fasted and reported for testing at the same time of day. Even though melatonin plays a role in modulating circadian rhythm patterns and GH is subject to diurnal variations, we standardized daily testing so that it was the same for all participants and followed previously-established guidelines [10-12]. Therefore, we are confident that the testing protocol was not a confounder of our results.

In males, 5.0 mg melatonin resulted in the greatest increase in serum GH during the pre-exercise period (157% increase from baseline), whereas in the post-exercise period both 0.5 mg (106% increase from baseline) and 5.0 mg (132% increase from baseline) melatonin effectively increased GH compared to placebo. Interestingly, during the pre-exercise period, we observed average (± SD) peak GH values to occur at 45 ± 4.5 and 40 ± 3.2 minutes, respectively, for the 0.5 mg and 5.0 mg groups. However, during the post-exercise period, we observed average peak GH values to occur at 25 ± 2.8 and 23 ± 2.2 minutes, respectively, for the 0.5 mg and 5.0 mg groups (Figure 2). Our GH results are not consistent with other data [10,13] that showed significant increases in GH in males with all melatonin doses. While Forsling et al. [13] did not involve an exercise bout, Meeking et al. [10] employed cycling exercise for 8 min at 70% of VO2_max_, and showed an increase in GH levels with 5.0 mg of melatonin when compared to placebo. More recently, however, 6 mg of oral melatonin in conjunction with a total-body resistance exercise bout involving 10 exercises, 25 total sets, and relative intensities of either 70% or 80% 1-RM was shown to have no effect in increasing in serum GH levels when compared to placebo [11].

Even though the present study was similar in design to a previous study [10], that reported no significant difference in GH levels between groups in the first 60 minutes following supplementation, our results for 5.0 mg melatonin suggest the contrary. However, it should be noted that unlike most studies, we measured free GH using an ultra-sensitive ELISA method in an effort to determine the levels of free, unbound GH without the detectable interference of circulating glycoproteins, GH-dependent peptide hormones, or GHBPs. Almost 50% of GH in circulation is bound to high affinity GHBPs, and circulating GHBP levels reflect the tissue GH receptor status due to the fact that GHBP arises from proteolytic cleavage of the extracellular domain of the GH receptor [14]. The fraction of bound GH decreases at or above a GH concentration in the middle of the physiological range [15], and acute resistance exercise has been shown to have no effect of the levels of serum GHBP [16]. As such, it is only the free, unbound GH which is able to bind to the extracellular domain of the GH receptor. Therefore, the determination of free GH is a much better representation of GH bio-availability resulting from melatonin supplementation and resistance exercise, and our results suggest both melatonin doses to increase free GH levels, particularly in males.

For GH in females, however, the response to melatonin supplementation was dissimilar to the response observed in males. While 0.5 mg melatonin was modestly increased during the post-exercise period (30% increase from baseline), the greatest increase in serum GH during both the pre- and post-exercise periods occurred in the placebo group (see Figure 2). While there are data demonstrating a significant increase in serum GH after an acute bout of resistance exercise in females, the increase was less than that observed in males [17].

As there have been few studies investigating the effects of oral melatonin in females, our present results are difficult to interpret. In the present study, we did not control for menstrual cycle activity; therefore, it is possible that the point of the 28-day menstrual cycle at which each female was tested could have led to the observed differences in serum GH. There are data demonstrating increases in serum GH levels from mid-cycle onward, despite only modest elevations in serum IGF-1, suggesting a lower sensitivity to GH in women [18]. The presence of gender differences in the GH/IGF-1 axis appears to be due to the level of endogenous estradiol, and suggests that estradiol affects GH action at the level of the GH receptor expression and function [19]. Women typically have higher mean 24-h GH levels than men as a result of greater GH secretory burst mass and a higher degree of disordered GH release [20], which may likely explain the higher GH levels in the placebo group in females, compared to males (Figure 1). However, it has been shown that for any given body surface area, the rate of disappearance of GH once in circulation reflect predominately the time mode of GH entry, rather than gender, menstrual cycle state, or prevailing estradiol concentration [21]. Therefore, while it is possible that females have a differential estradiol-induced responsiveness in GH release, it is unlikely that menstrual cycle activity (and our lack of control of it in the present study) played a role in our results.

In both males and females, serum free IGF-1 levels did not significantly increase in response to melatonin supplementation or resistance exercise and there were no significant differences between supplements (Figure 3). Dall et al. [22] observed no changes in free IGF-1 following a maximal treadmill test lasting seven min in duration. While any increases in serum GH are short-lived, the subsequent GH-mediated hepatic IGF-1 release in response to resistance exercise may take anywhere from 8–30 hours, and any immediate increase in serum IGF-1 levels is typically due to its release from disrupted cells already containing IGF-1 [23]. A high-intensity bout of resistance exercise in men was shown to significantly increase serum GH; however, this response did not appear to affect IGF-1 levels after a 24-h recovery period [24].

Relative to IGFBP-1, our results support those of Meeking et al. [10] and we observed no significant increase in response to melatonin supplementation or resistance exercise for both males and females (Figure 4). IGFBP-1, which is thought to be a direct regulator of free IGF-1, increases during states of catabolism [25]. As such, the levels of IGFBP-1 seem to be unresponsive to exercise less than 30 min in duration, whereas longer duration exercise significantly increases IGFBP-1 [26]. IGFBP-1 appears to be associated with basal GH secretion rate [27], and has also been shown to be down-regulated by circulating insulin [28] and GH levels [29]. Even though we did not measure insulin in this study we measured serum glucose levels with our serum clinical chemistry panel and discovered that there were no significant changes between supplements throughout the testing session. Furthermore, Meeking et al. [10] also showed no significant difference between groups for serum insulin or glucose. Therefore, our observations can allow us to safely assume that there was likely not a significant insulin response and hence no change in IGFBP-1 that may have otherwise been mediated by melatonin supplementation or resistance exercise.

For males, 0.5 mg and 5.0 mg melatonin resulted in notable increases in serum IGFBP-3 during both the pre- (0.5 mg and 5.0 mg = 8% and 6% increases from baseline, respectively) and post-exercise periods (0.5 mg and 5.0 mg = 11% and 9% increases from baseline, respectively) that were not significant from each other or placebo. Although not significant, levels were even greater during the post-exercise period suggesting IGFBP-3 to be responsive to resistance exercise. In females, modest increases in IGFBP-3 were observed in all three supplements during the pre-exercise period, with the placebo and 5.0 mg melatonin groups exhibiting further increases during the post-exercise period (see Figure 5). Since IGFBP-3 increases concomitant with GH, for males our data portray this trend in both the 0.5 mg and 5.0 mg melatonin doses. Previous studies have shown increases in IGFBP-3 following acute maximal exercise with protocols involving a treadmill [22] and cycle ergometer [30]. IGFBP-3 is primarily involved in transporting most (>75%) circulating IGF-1 to the muscle where it binds to the IGF-1 receptor [31]. The proteolytic activity of IGFBP-3 may be a potent regulator of IGF-1 bioactivity [32], and increases in IGFBP-3 proteolysis would release bound IGF-1 thereby increasing the circulating levels of free IGF-1. It has been suggested that increases in IGFBP-3 are correlated with IGFBP-3 proteolysis; however, Schwarz et al. [30] demonstrated that IGFBP-3 proteolytic activity was not affected by an acute bout of maximal cycling exercise. In the present study, we observed increases in IGFBP-3 but no increases in free IGF-1, suggesting no proteolytic activity of IGFBP-3. Therefore, based on our results this could indicate a possible mechanism by which melatonin instigates elevations in IGFBP-3 release that are not associated with IGFBP-3 proteolysis and the subsequent release of bound IGF-1.

In males, 5.0 mg melatonin resulted in the greatest decrease in serum SST during the pre-exercise period (164% increase from baseline) while 0.5 mg melatonin decreased 70% from baseline, whereas in the post-exercise period both 0.5 mg (44% decrease from baseline) and 5.0 mg (76% decrease from baseline) melatonin effectively decreased SST compared to placebo. Interestingly, during the pre-exercise period, we observed average (± SD) peak decreases in SST values to occur at 28 ± 5.7 and 33 ± 6.4 minutes, respectively, for the 0.5 mg and 5.0 mg groups. However, during the post-exercise period, we observed average peak decreases in SST values to occur at 14 ± 3.6 and 19 ± 4.2 minutes, respectively, for the 0.5 mg and 5.0 mg groups (Figure 6).

For SST in females, the response to melatonin supplementation was similar to the response observed in males; however, the magnitude of the response was significantly less. During the pre-exercise period, 0.5 and 5.0 mg melatonin were decreased 35% and 47%, respectively, from baseline. While 5.0 mg melatonin was modestly decreased during the post-exercise period (32% increase from baseline), 0.5 mg melatonin only decreased 14% from baseline (Figure 6).

Based on the pre-exercises responses in serum GH for males for 5.0 mg melatonin, our data support the contention that melatonin increases serum GH levels due to an apparent inhibition of SST. However, the post-exercise responses for serum GH were greater with 0.5 mg melatonin while SST was less than during the pre-exercise period, which suggest the potential interactive effects of resistance exercise. The underlying mechanisms for exercise-induced increases in GH are not well known. Many factors such as the mode, duration, and intensity of exercise, age, gender, body composition, and nutritional and training status may modulate increases in GH [33]. In the present study, we controlled for all of these afore-mentioned variables in such a way that our results are primarily contingent on the melatonin supplement and/or resistance exercise. The increase in GH release in response to sub-maximal exercise is mainly mediated by an increased central cholinergic tone [34], which reduces the activity of hypothalamic SST. Increased GH release from high-intensity exercise, however, appears to likely be potentiated from the partial inhibition of SST as well as the concomitant activation of endogenous GHRH [35]. Based on the observed patterns of response from the 0.5 mg and 5.0 mg melatonin doses in the post-exercise period, this scenario is conceivable.

For hemodynamic and serum and whole blood clinical safety markers, none of the variables measured were shown to be changed throughout the course of the testing session for all three supplements (Tables 1, 2, 3). Therefore, within the confines of the experimental design, our data suggests that melatonin is safe to supplement orally based on the hemodynamic safety markers, and the serum and whole blood clinical chemistry markers we assessed. In addition, there were also no adverse side effects from ingesting the melatonin supplements reported by the subjects immediately following the testing session.

In conclusion, for males 5.0 mg melatonin appears to increase serum GH while concomitantly lowering SST levels; however, when combined with resistance exercise both 0.5 mg and 5.0 mg melatonin appears to positively impact GH levels in a manner not entirely dependent on decreases in SST. Relative to the response in females, it appears that both melatonin and resistance exercise elicits a greater GH response in males.

## Competing interests

The author(s) declare that they have no competing interests.

## Authors' contributions

EN and CM participated in the design of the study, coordination and data acquisition, and assisted in performing the statistical analysis and drafting the manuscript. LT, CK, and MG participated in the data acquisition. MG participated in the design of the study and assisted in performing the statistical analysis. RK assisted with the design of the study, in securing funding for the project, and in the statistical analysis. DSW conceived the study, developed the study design, secured the funding for the project, assisted and provided oversight for all data acquisition and statistical analysis, assisted in drafting the manuscript, and served as the faculty mentor for the project. All authors have read and approved the final manuscript.

## References

[B1] Hindmarsh P, Matthews D, Brain C, Pringle P, di Silvio L, Kurtz A, Brook C (1989). The half-life of exogenous growth hormone after suppression of endogenous growth hormone secretion with somatostatin. Clin Endocrinol.

[B2] Mullis P, Wagner J, Eble A, Nuoffer J, Postel-Vinay M (1997). Regulation of human growth hormone receptor gene transcription by human growth hormone binding protein. Mol Cell Endocrinol.

[B3] Vanhelder W, Radomski M, Goode R (1984). Growth hormone responses during intermittent weight lifting exercise in men. Eur J Appl Physiol.

[B4] Goto K, Sato K, Takamatsu K (2003). A single set of low intensity resistance exercise immediately following high intensity resistance exercise stimulates growth hormone secretion in men. J Sports Med Phys Fitness.

[B5] Izquierdo-Claros R, Boyano-Adanez M, Torrecillas G, Rodriquez-Puyol M, Arilla-Ferreiro E (2001). Actue modulation of somatostatin receptor function by melatonin in the rat frontoparietal cortex. J Pineal Res.

[B6] Valcavi R, Dieguez C, Azzarito C, Edwards C, Dotti C, Page M, Portioli I, Scanlon M (1987). Effect of oral administration of melatonin on GH responses to GRF 1–44 in normal subjects. Clin Endocrinol (Oxf).

[B7] Coiro V, Volpi L, Capretti N, Giuliani G, Caffarri R, Colla C, Marchesi C, Chiodera P (1998). Different effects of naloxone on the growth hormone response to melatonin and pyridostigmine in normal men. Metabolism.

[B8] Molinari E, North P, Dubocovich M (1996). 2-[125I]iodo-5-methoxycarbonylamino-N-acetyltryptamine: a selective radioligand for the characterization of melatonin ML2 binding sites. Eur J Pharmacol.

[B9] Valcavi R, Zini M, Maestroni G, Conti A, Portioli I (1993). Melatonin stimulates growth hormone secretion through pathways other than the growth hormone-releasing hormone. Clin Endocrinol (Oxf).

[B10] Meeking D, Wallace J, Cuneo R, Forsling M, Russell-Jones D (1999). Exercsie-induced GH secretion is enhance by the oral ingestion of melatonin in health adult male subjects. Eur J Endocrinol.

[B11] Mero A, Vahalummukka M, Hulmi J, Kallio P, von Wright A (2006). Effects of resistance exercise session after oral ingestion of melatonin on physiological and performance responses of adult men. Eur J Appl Physiol.

[B12] Maas H, deVries W, Maitimu I, Bol E, Bowers C, Koppeschaar H (2000). Growth hormone responses during strenuous exercise: the role of GH-releasing hormone and GH-releasing peptide-2. Med Science Sports Exerc.

[B13] Forsling M, Wheeling M, Williams A (1999). The effect of melatonin administration on pituitary hormone secretion in man. Clin Endocrinol.

[B14] Zafeiridis A, Smilios I, Considine R, Tokmakidis S (2002). Serum leptin responses after acute resistance exercise protocols. J Appl Physiol.

[B15] Baumann G, Amburn K, Shaw M (1988). The circulating growth hormone (GH)-binding protein complex: a major constituent of plasma GH in man. Endocrinology.

[B16] Rubin M, Kraemer W, Maresh C, Volek J, Ratamess N, Vanheest J, Silvestre R, French D, Sharman M, Judelson D, Gomez A, Vescovi J, Hymer W (2005). High-affinity growth hormone binding protein and acute heavy resistance exercise. Med Sci Sports Exerc.

[B17] Linnamo V, Pakarinen A, Komi P, Kraemer W, Hakkinen K (2005). Acute hormonal responses to submaximal and maximal heavy resistance and explosive exercises in men and women. J Strength Cond Res.

[B18] Gleeson H, Shalet S (2005). GH responsiveness varies during the menstrual cycle. Eur J Endocrinol.

[B19] Leung K, Johannsson G, Leong G, Ho K (2004). Estrogen regulation of growth hormone action. Endocr Rev.

[B20] Van den Berg G, Veldhuis J, Frolich M, Roelfsema F (1996). An amplitude-specific divergence in the pulsatile mode of growth hormone (GH) secretion underlies the gender difference in mean GH concentrations in men and premenopausal women. J Clin Endocrinol Metab.

[B21] Shah N, Aloi J, Evans W, Veldhuis J (1999). Time mode of growth hormone (GH) entry into the bloodstream and steady-state plasma GH concentrations, rather than sex, estradiol, or menstrual cycle stage, primarily determine the GH elimination rate in healthy young women and men. J Clin Endocrinol Metab.

[B22] Dall R, Henrik K, Longe W, Kjaer M, Otto J, Jorgensen L, Christansen J, Orskov H, Flyvbjerg A (2001). No evidence of insulin-like growth factor-binding protein 3 proteolysis during a maximal exercise test in elite athletes. J Clin Endocrinol Metab.

[B23] McArdle W, Katch F, Katch V (2001). Exercise Physiology: Energy, Nutrition, and Human Performance.

[B24] Kraemer W, Aguilera B, Terada M, Newton R, Lynch J, Rosendaal J, McBride J, Gordon S, Hakkinen K (1995). Responses of IGF-I to endogenous increases in growth hormone after heavy-resistance exercise. J Appl Physiol.

[B25] Lindgren B, Odar-Cederlof F, Ericsson F, Brismar K (1996). Decreased bioavailability of insulin-like growth factor-1, a cause of catabolin in hemodialysis patients. Growth Regul.

[B26] Nguyen U, Mougin F, Simon-Riguard M, Rouillon J, Marguet P, Regnard J (1998). Influence of exercie duration on serum insulin-like growth factor and its binding proteins in athletes. Eur J Appl Physiol Occup Physiol.

[B27] Hansen T, Jorgensen J, Christiansen J (2002). Body composition and circulating levels of insulin, insulin-like growth factor-binding protein-1 and growth hormone (GH)-binding protein affect the pharmacokinetics of GH in adults independently of age. J Clin Endocrinol Metab.

[B28] Cichy S, Uddin S, Danilkovich A, Guo S, Klippel A, Unterman T (1998). Protein kinase B/Akt mediates effects of insulin on hepatic insulin-like growth factor-binding protein-1 gene expression through a conserved insulin response sequence. J Biol Chem.

[B29] Norrelund H, Fisker S, Vahl N, Borglum J, Richelsen B, Christiansen J, Jorgensen J (1999). Evidence supporting a direct suppressive effect of growth hormone on serum IGFBP-1 levels. Experimental studies in normal, obese and GH-deficient adults. Growth Horm IGF Res.

[B30] Schwarz A, Brasel J, Hintz R, Mohan S, Cooper D (1996). Actue effect of brief low- and high-intensity exercise on circulating insulin-like growth factor (IGF) I, II, and IGF-binding protein-3 and its proteolysis in young and health men. J Clin Endocrinol Metab.

[B31] Kraemer R, Durand R, Acevedo E, Johnson L, Kraemer G, Hebert L, Castracane V (2004). Rigorous running increases growth hormone and insulin-like growth factor-I without altering ghrelin. Exp Biol Med.

[B32] Fowlkes J (1997). Insulin-like growth factor binding protein proteolysis. Trends Endocrinol Metab.

[B33] Cuneo R, Wallace J (1994). Growth hormone, insulin-like growth factors and sport. Endocrinol Metab.

[B34] Nooitgedagt A, Koppeschaar J, de Vries W, Sidorowicz A, Klok L, Dieguez C, Mallo F (2002). Influence of endogenous cholinergic tone and growth hormone-releasing peptide-6 on exercise induced growth hormone release. Clin Endocrinol.

[B35] DeVries W, Abdesselam S, Schers T, Maas H, Osman-Dualeh M, Maitimu I, Koppeschaar H (2002). Complete inhibition of hypothalamic somatostatin activity is only partially responsible for the growth hormone response to strenuous exercise. Metabolism.

